# Assessing contraband tobacco in two jurisdictions: a direct collection of cigarette butts

**DOI:** 10.1186/s12889-016-3229-0

**Published:** 2016-07-22

**Authors:** Julie Stratton, Samantha Shiplo, Megan Ward, Alexey Babayan, Adam Stevens, Sarah Edwards

**Affiliations:** Peel Public Health, 7120 Hurontario Street, Mississauga, ON L5W 1N4 Canada; Brant County Health Unit, 194 Terrance Hill Street, Brantford, ON N3R 1G7 Canada

**Keywords:** Tobacco products, Contraband tobacco, Smoking behaviours, Smoking-prevention and control, Tobacco industry, Unobtrusive observation, Public health

## Abstract

**Background:**

The sale of contraband tobacco allows for tobacco tax evasion, which can undermine the effectiveness of tobacco tax policies in reducing the number of smokers. Estimates of the proportion of contraband vary widely as do the methods used to measure the proportion of contraband being smoked. The purpose of this study is to determine the proportion of contraband use in two different jurisdictions.

**Methods:**

A cross-sectional direct collection of cigarette butts was conducted in Peel and Brantford, Ontario, Canada in 2013 and 2014, respectively. Cigarette butts were collected from a variety of locations within both regions. Cigarette butts were assessed and classified into one of the following categories: contraband, legal Canadian, legal Native, International, unknown, and discards.

**Results:**

The overall proportion of contraband cigarettes in Peel was 5.3 %, ranging from 2.8 to 8.6 % by location. In Brantford, the proportion of contraband was 33.0 %, with a range from 32.8 to 33.1 % by location.

**Conclusions:**

The direct collection of cigarette butts was determined to be a feasible method for a local public health unit in determining the proportion of contraband cigarettes. This approach showed that Brantford has a higher proportion of contraband consumption compared to Peel, which may be due to geographic location and proximity to the United States (US)-Canada border and Native Reserves. More research is needed to confirm this geographic association with other jurisdictions.

## Background

Smoking, along with other forms of tobacco use, remains the leading cause of preventable mortality and morbidity in Ontario, Canada, resulting in over 13,000 deaths and $6 billion in direct health care expenditures and lost productivity each year [1]. Ontario has made substantial progress in reducing the rates of smoking among youth and adults and minimizing the exposure to second-hand smoke; however, tobacco use continues to be a public health concern. Along with persistent socio-demographic variability in tobacco use and low rates of tobacco taxation compared to the rest of Canada, the presence of contraband tobacco has been recognized as a potential barrier to curbing tobacco use in Ontario [2].

Contraband tobacco is defined as any tobacco product that does not comply with the provisions of all applicable federal and provincial statutes, including importation, stamping, marking, manufacturing, distributing, and payment of duties and taxes [3]. Purchasing contraband tobacco, or taking part in tobacco tax evasion, is likely to impact smoking cessation efforts. For instance, an international study found that smokers were 28 % less likely to quit smoking if they engaged in price minimization or tax avoidance behaviours [4]. Lower priced contraband tobacco can reduce the positive impact of taxation on decreasing smoking prevalence and undermine public health efforts to reduce tobacco-related morbidity and mortality [5, 6]. Populations of particular concern are youth, low income earners, and socially deprived groups, which have been shown to be sensitive to changes in tobacco pricing via taxation [7–11]. As a rule, contraband is less expensive to purchase. A package of about 200 untaxed cigarettes sells for approximately six dollars per carton compared to $75 to $90 dollars per carton for taxed cigarettes [3].

In Canada, the main sources of contraband tobacco come from: products that are unlawfully manufactured in Canada or the US, or lawfully manufactured in the US and smuggled into Canada; diverted tax-exempt products; international brands entering Canada illegally by sea container; stolen tobacco products that are resold; and tobacco sales through the internet where duties or taxes are not paid [3, 12, 13]. Since 2001, most contraband tobacco has originated from manufacturing operations based on First Nation reserves and territories on either side of the US and Canadian border, including the Kahnawake reserve in Quebec; the Tyendinaga and Six Nations reserves in Ontario, and the Akwesasne reserve in the US [13, 14].

The methods used to measure the proportion of contraband being smoked vary widely. Self-report surveys, direct collection of dropped cigarette packages, direct observation of possessed cigarette packages, and direct collection of cigarette butts are previously used methods for measuring contraband tobacco.

In Canada and Ontario, several population-based surveys have been conducted using a variety of questions to measure contraband cigarette use, such as self-reported purchasing of contraband cigarettes and use of contraband cigarettes [15–17].

In 2007-08, the Ontario Tobacco Survey found that 29 % of total reported cigarette consumption among current smokers in Ontario was contraband cigarettes bought on reserves [18]. Data from the 2006-07 Youth Smoking Survey indicates that approximately 9 % of Canadian youth (grades 6 to 12) were smoking contraband cigarettes and that these youth were more likely to be heavy smokers with limited spending money [19]. Results from the Ontario Student Drug Use and Health Survey (OSDUHS) show that the proportion of Ontario students smoking Native brand “contraband” cigarettes decreased from 6 to 3 % between 2009 and 2013 [16]. These population-based studies are based on self-report. While they have the advantage of relatively fast administration, they can be affected by social desirability bias and possible under-reporting, especially among youth [20].

Studies involving collection of dropped cigarette packages have been used to estimate tax avoidance. This type of study is unobtrusive and can provide a measure of tax avoidance [21–23]. However, it could provide biased estimates, specifically related to the behaviours of a person who litters and one who does not [21].

Studies using the direct observation of possessed cigarette packages have been conducted in several countries. The methodology used in this type of study involves the recruitment of smokers to provide their cigarette pack to researchers; this adds more complexity to the study design and sampling strategy [24, 25].

Evidence of the use of contraband cigarettes is also available from direct cigarette butt collection [26]. In this method, every butt within a specified area is collected and sorted into legitimate and contraband groups. The proportion of contraband can then be precisely estimated. Direct cigarette butt collection is considered feasible since the littering of cigarette butts is ubiquitous in the environment. Furthermore, the direct collection of cigarette butts can provide an explicit estimate of the proportion of contraband cigarettes being smoked by location. This methodology is unobtrusive and avoids the social desirability bias that may be present in self-reported surveys [26]. The Canadian Convenience Store Association commissions ad hoc cigarette butt studies and in 2009, suggested that 30 % of cigarette butts collected from 110 sites in Ontario were contraband; however, limited information is available regarding the methodology and sampling used in this study [27]. A study by Barkans and Lawrence [26], which focused on young adults, examined over 36,000 dropped cigarette butts collected from 25 campuses purposively selected across Ontario [26]. This study found that 14 % of collected cigarette butts were contraband, and that the prevalence varied greatly by region (2 to 39 %). In 2013, the Ontario Convenience Store Association (OCSA) analyzed 18,000 cigarette butts collected from 136 public smoking locations in Ontario and reported that 21 % of collected cigarette butts were contraband [28]. Overall, cigarette butt studies have arrived at different estimates of contraband tobacco consumption across Canada and in Ontario, which may in part be explained by differences in methodologies employed and locations of the studies. Nevertheless, these studies suggest that the proportion of contraband may vary by population groups and regions.

In the current study, the primary objective was to determine the proportion of contraband cigarettes smoked in two Canadian jurisdictions: a large municipality and a medium-sized community. The secondary objective was to test the methods for direct cigarette butt collection to determine if they were feasible for a local public health department.

With a population of almost 1.4 million people, Peel Region is a large, upper tier municipality in Ontario, which is comprised of the cities of Mississauga and Brampton, and the Town of Caledon. In 2014, 12.0 % (95 % CI: 9.1–14.9 %) of Peel’s residents aged 12 years and older were current smokers [29]. In 2012, Peel Regional Council received a written request from the Ontario Convenience Store Association (OCSA) advocating that the Region write to the Ontario Ministry of Finance asking for the problem of contraband to be addressed. The OCSA reported that contraband cigarette use among youth was as high as 44 % in Peel region schools. Given the lack of transparency in the methods to conduct the OSCA study, Peel was interested in testing a methodological approach to determining the proportion of contraband cigarette butts in the region.

Brantford is a medium-size community in Southwestern Ontario with a population of 93,650 people. Tobacco use remains a public health concern in this community. In Brantford, 29.0 % (95 % CI: 21.1–36.9 %) of the population aged 12 years and older were current smokers in 2014, which is significantly higher than the rate of smoking across Ontario as a whole (17.4 %; 95 % CI: 16.5–18.3 %) [29]. While the prevalence of smoking in the province has been decreasing over the past 10 years, the rate of smoking in Brantford has remained unchanged [29]. Easy access to contraband tobacco may be one of the factors contributing to high rates of smoking [30]. The city of Brantford borders the largest First Nation reserve in Canada, and is alleged to have several unlicensed cigarette factories [14].

Determining the proportion of contraband tobacco use through a direct cigarette butt collection methodology in Peel Region and the City of Brantford was designed to help both jurisdictions understand the magnitude of the issue and inform decision making around public health strategies aimed at reducing tobacco use.

## Methods

### Sampling frame and sample size

Study locations were purposefully selected in both jurisdictions. In Peel, both regional buildings serving the public and all three hospital sites were selected to represent the general population, all three post-secondary campuses were selected to represent young adults, and 10 secondary schools out of 67 were randomly selected to represent youth. In Brantford, six public places were selected to represent the general population (including an administrative services building, an outdoor recreation plaza, and a bus terminal, general hospital, a sports center, and a mall). All five secondary schools were selected to represent the youth population. At the time of data collection, there were no active post-secondary campuses in Brant, therefore this type of venue was excluded from the sampling frame. Administrative approval for collection was obtained.

The process for cigarette butt collection and identification was piloted at one site from each type of location prior to conducting the full study in order to validate the process. This also helped to confirm the percent of contraband smoked by location which was required for final sample size estimation.

The sample size was calculated using the following parameters: the percent of contraband cigarette butts obtained from the pilot study (6 % in Peel; 32.5 % in Brantford); a design effect of 1/0.7 to adjust for the fact that cigarette butts collected from the same sites are more alike [31]; a desired precision of the estimate to detect differences between locations (3 %); statistical power of 80 %; type 1 error rate of 5 %; and discard rate by venue as determined from the pilot study. Based on these factors and assumptions, we estimated that a total of 6,094 cigarette butts would be required from the four types of locations in Peel Region, and a total of 12,354 cigarette butts from the two types of locations (public places and secondary schools) in Brantford to ensure sufficient power and precision for estimating the prevalence of contraband cigarette consumption in both jurisdictions [32].

### Data collection

The study was approved by the ethics review boards of the Peel Public Health and the Brant County Health Unit. Institutions in each of the selected sites were notified about the purpose, procedure, and timeline of the study, and a signed consent form was obtained, via e-mail or fax, prior to the start of data collection. Data collection occurred between August 16 and September 26, 2013 in Peel region and from August 18 to September 23, 2014 in Brantford.

Collection depended on weather conditions as cigarette butts could only be collected on dry days. At each site, all cigarette butts within the specified area were collected from cigarette butt receptacles and the ground. For sites without cigarette butt receptacles, all cigarette butts were collected from the ground.

### Data analysis

Trained research assistants in each jurisdiction examined and classified cigarette butts into one of six groups: contraband, legal native, legal Canadian, international, unknown, and discard. Contraband cigarettes are illegally manufactured or sold. Legal native cigarettes are manufactured and sold by First Nations owned tobacco companies holding a tobacco license [3, 33]. Legal Canadian cigarettes are sold by non-First Nations, Canadian tobacco companies holding a tobacco license. International cigarettes are those manufactured and sold outside of Canada. Unknown cigarettes are those that could not be identified. In addition, some of the collected cigarette butts were classified as discards as they were too damaged or too small for examination and classification. All examined cigarette butts were counted and documented.

Cigarette butts were classified based on brand labelling or other identifiable markings on the cigarette tipping paper (Table 1). To ensure that cigarette butts were not wrongfully classified, the research team consulted a cigarette brand database, www.cigarettespedia.com, and government organizations doing similar work.

**Table 1:** Classification of cigarette butts

Category	Legal Canadian	Contraband	Legal native	International
Review brand name or symbol	• Player’s • DuMaurier • Belmont • Peter Jackson • Vogue, etc.	• Play Fare • CANADIAN • Deerfield • disCOUNT • NF • TMT • or coloured banding, etc.	• DK’s • Putters • Podium • Ménage • Sago	• Richmond • LD • XL • Castor • Fortune • Parliament, etc.
Presence of a dry patch	Usually present	Not usually present	Not usually present	May or may not be present
Presence of ventilation holes	Present	Usually present	Not usually present	May or may not be present
Type of filter (acetate or polypropylene)	Acetate filter	May have polypropylene filter	Usually have acetate filter	

**Fig. 1:**
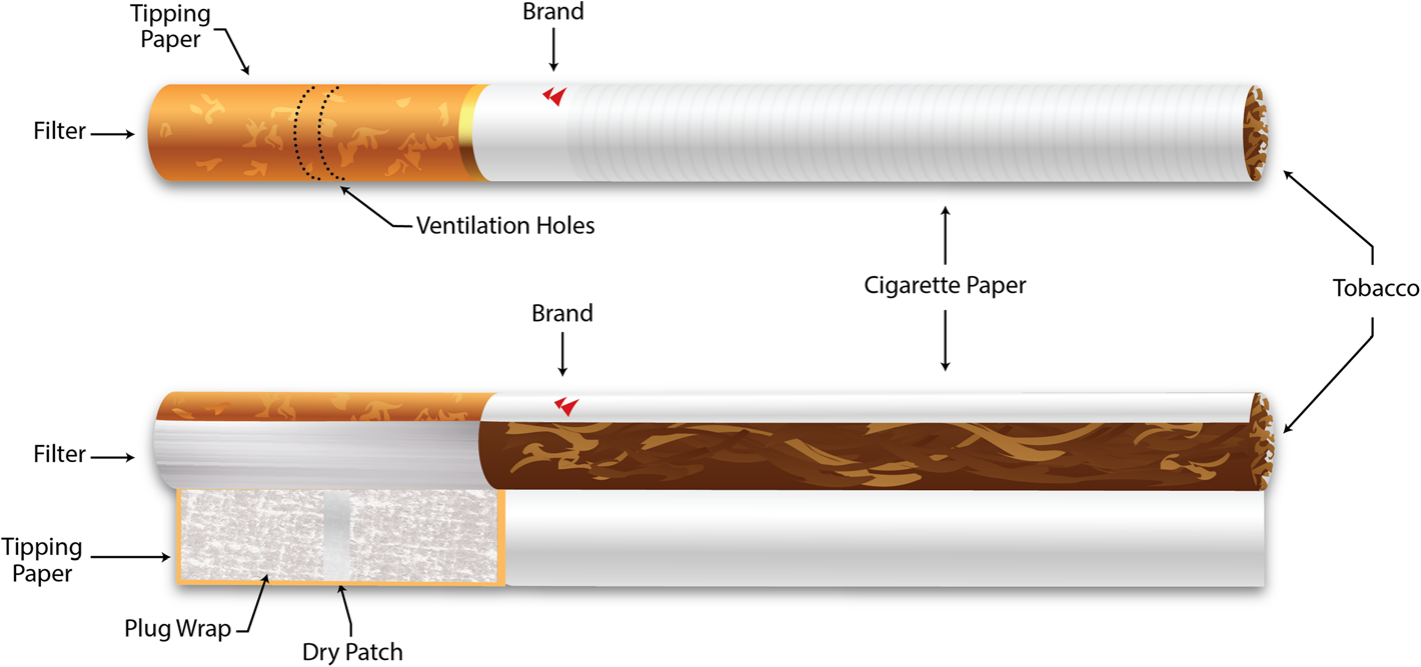
Anatomy of a Cigarette. Source: Peel Public Health

Furthermore, cigarette butts were cut open and tested to assess the following: presence of a dry patch, ventilation holes (ie, the number of rows of ventilation holes and whether the holes were made mechanically, with a laser, or electromagnetically), and filter composition (ie, acetate or polypropylene). The dry patch is in the middle of the plug wrap where there is no glue. To assess whether a dry patch is present, a small amount of water or iodine can be placed on the plug wrap to make the area more visible. Ventilation holes are found on the butt end of the cigarette and are intended to allow air flow into the cigarette while it is being smoked. Ventilation holes can be made by electrostatic perforation, laser perforation, and mechanical perforation. Furthermore, there are two types of filters used in cigarettes: polypropylene and acetate. To assess filter composition, the filter was removed and placed into acetone. Acetate dissolves in acetone, while polypropylene does not. Figure 1 depicts the components of the cigarette that were assessed for identification.

Descriptive statistics were used to identify proportions of cigarette butts in each classification and 95 % confidence intervals (95 % CI) were calculated based on the binomial distribution. Differences in the proportion of contraband cigarettes observed between locations were assessed using a chi-square test in Open-Epi (http://www.openepi.com/TwobyTwo/TwobyTwo.htm).

## Results

A total of 14,032 cigarette butts were collected in Peel and 12,918 cigarette butts were collected in Brantford, exceeding our sample size requirements. In Peel and Brantford respectively, 14.6 % (2,051) and 16.5 % (2,134) of the total cigarette butts collected were discarded because they were too damaged to be categorized. As a result, a total of 11,981 cigarette butts in Peel and 10,784 cigarette butts in Brantford were included in the final analysis. In Peel, the majority of cigarettes (89.0 %) were classified as legal Canadian cigarettes and only 5.3 % were classified as contraband. In Brantford, 40.1 % of cigarettes collected were classified as legal Canadian and 33.0 % as contraband (Table 2).

**Table 2:** Distribution of cigarette butt type, Peel Region, 2013 and City of Brantford, 2014

Classification	Peel		Brantford	
	N^a^	%	N^a^	%
Legal Canadian	10,663	89.0	4,323	40.1
Contraband	630	5.3	3,558	33.0
Legal Native	530	4.4	2,705	25.1
International	144	1.2	190	1.8
Unknown	14	0.1	8	0.1
Total	11,981	100.0	10,784	100.1

^a^Discards cigarette butts excluded: Peel region, *n* = 2,051; Brantford, *n* = 2,134

Peel secondary schools, reflecting youth smokers, had the highest proportion of contraband cigarettes (8.6 %) compared to college and university campuses (2.8 %); and hospitals and regional buildings (5.5 %). In Brantford, secondary schools and sites representing the general public had a similar proportion of contraband cigarettes (32.8 % and 33.1 %) (Table 3).

**Table 3:** Proportion of contraband cigarette butts by population groups, Peel Region, 2013 and City of Brantford, 2014

Population groups (Sites)	Peel		Brantford	
	n	% (95 % CI)	n	% (95 % CI)
General population (Hospitals, Regional buildings, Recreation plaza^a^, Bus station^a^, Sports Centre^a^, Mall^a^)	280	5.5 (4.9–6.1)	2,677	33.1 (32.0–34.1)
Youth (Secondary Schools)	235	8.6 (7.6–9.8)	881	32.8 (31.0–34.5)
Young adult (College/University)	115	2.8 (2.3–3.3)	N/A	N/A
Total	630	5.3 (4.9–5.7)	3,558	33.0 (32.1–33.9)

^a^Brantford only sites

*N/A* data not available

## Discussion

This study describes the proportion of contraband cigarette butts determined through the direct collection of cigarette butts. Among the cigarette butts collected from a sample of locations in Peel region and Branford, 5 and 33 % respectively were contraband. Given that the same methodologies for direct cigarette butt collection were used in both areas, the difference in estimates observed between these jurisdictions suggests that geographic location may be contributing to the high proportion of contraband cigarettes in Brantford.

Geographic location is an important predictor of smoking, even after accounting for individual, socioeconomic, and demographic characteristics [34]. Brantford borders a large First Nations reserve, where a number of unlicensed cigarette factories are reportedly located [14]. Although there is no direct evidence regarding access of the Brantford population to contraband cigarettes from the neighboring First Nation community, in general, First Nations reserves have been recognized as a primary source of the contraband tobacco market in Canada [13]. The application of the study methods into other jurisdictions across Ontario and Canada would provide more insight into contraband consumption by geography as well as proximity to contraband cigarettes outlets.

The finding that 8.6 and 32.8 % of cigarette butts collected from secondary schools in Peel and Brantford respectively were contraband, suggests a high proportion of contraband consumption among youth smokers in both jurisdictions. Given that youth are sensitive to changes in tobacco pricing, the observed higher contraband consumption among youth may be explained by the reduced cost barriers due to the availability of cheap contraband tobacco. Thus, targeting public health efforts to the youth population is essential when addressing the issue of contraband cigarette use in the community [19].

The present study has a number of strengths. The study involved a direct, unobtrusive collection of cigarette butts, avoiding potential underestimation of contraband use that typically occurs in self-reported surveys. Additionally, this study had a large sample size, a wide variety of locations which captured cigarette use among the youth, young adult, and the general populations; and external validation of the classification of unbranded and non-Canadian cigarettes. The study methodology and protocols can be easily replicated if there is a need to understand the magnitude of contraband cigarette smoking in other regions or communities.

There are several limitations of this study that need to be acknowledged. In this study, we opted for locations with high public traffic. While there are many other types of locations that could have been selected (eg, such as restaurants, bars), there is no literature to suggest that the proportion of contraband tobacco smoked in the locations we selected is different from contraband tobacco smoked in other public locations. In addition, the venues selected to represent the general population in the two jurisdictions differed; however, since the same population was served we feel the building type should not bias the results.

Given the variation in contraband cigarette use by geography found in this study and results from only two locations within Ontario, the study has limited generalizability to other jurisdictions. For this reason, the study results were not pooled. Furthermore, the study only examined the proportion of contraband cigarettes smoked, while the proportion of individuals using contraband cannot be inferred from this data. Finally, misclassification of cigarette butts could have occurred in some instances such as with roll-your-own cigarettes and international cigarettes. The potential for misclassification of roll-your-own cigarettes results from two factors; roll-your-own cigarette machines which produce professionally manufactured cigarettes; and the paper sold for roll- your-own cigarettes which make them look similar to contraband. Given that only 9.8 % of current smokers in Canada used roll-your-own cigarettes in 2010, and that this statistic is not reportable in Ontario due to small numbers, we do not feel that this would have biased our results [35]. It is also possible that cigarette butts classified as legal international and legal native may have been obtained illegally without taxes paid; however, this could not be determined in the current study.

Overall, the study’s findings suggest that contraband tobacco use is not a significant issue in Peel; however, it demonstrates that contraband tobacco use is common in Brantford, which may undermine cessation efforts and reinforce smoking behavior in that community. Smokers who use contraband cigarettes are most likely to continue doing so [36] and are less likely to quit smoking [4]. Thus, approaches to reduce smoking rates may differ between Peel and Brantford.

Previous research has provided recommendations regarding strategies to reduce contraband consumption, including increased law enforcement and public education campaigns targeted to specific populations of smokers such as those representing different age and socio-economic groups [26]. Furthermore, cooperation with First Nations on law enforcement and their engagement in development of public education messages would be necessary to ensure success of the strategies and overall efforts to reduce availability and use of contraband tobacco [37].

## Conclusion

This study utilized the direct collection of cigarette butts in order to determine the proportion of contraband cigarettes in each jurisdiction. Our results suggest that geographic location may be contributing to the high proportion of contraband cigarettes in one of the sampled jurisdictions. Additional studies using this methodology in different jurisdictions are needed. Additionally, the higher rate of contraband cigarettes found in locations frequented by youth suggest that the lower price of contraband cigarettes may be encouraging the uptake of smoking among youth.

## Abbreviations

OCSA, Ontario Convenience Store Association; OSDUHS, Ontario Student Drug Use and Health Survey; US, United States
